# Supplementary material for: Cooperative growth in microbial communities is a driver of multistability
Source: Nat Commun. 2024 Jun 3;15:4709. doi: 10.1038/s41467-024-48521-9 (PMC11148146; doi:10.1038/s41467-024-48521-9)
Supplement: Supplementary file 1 — Supplementary Information [file 41467_2024_48521_MOESM1_ESM.pdf]

Supplementary information for

Cooperative growth in microbial communities is a driver of multistability

William Lopes, Daniel R. Amor & Jeff Gore
Correspondence to: gore@mit.edu; lopesw@mit.edu

This PDF file includes:

Supplementary Figures 1 to 11
Supplementary Text 1

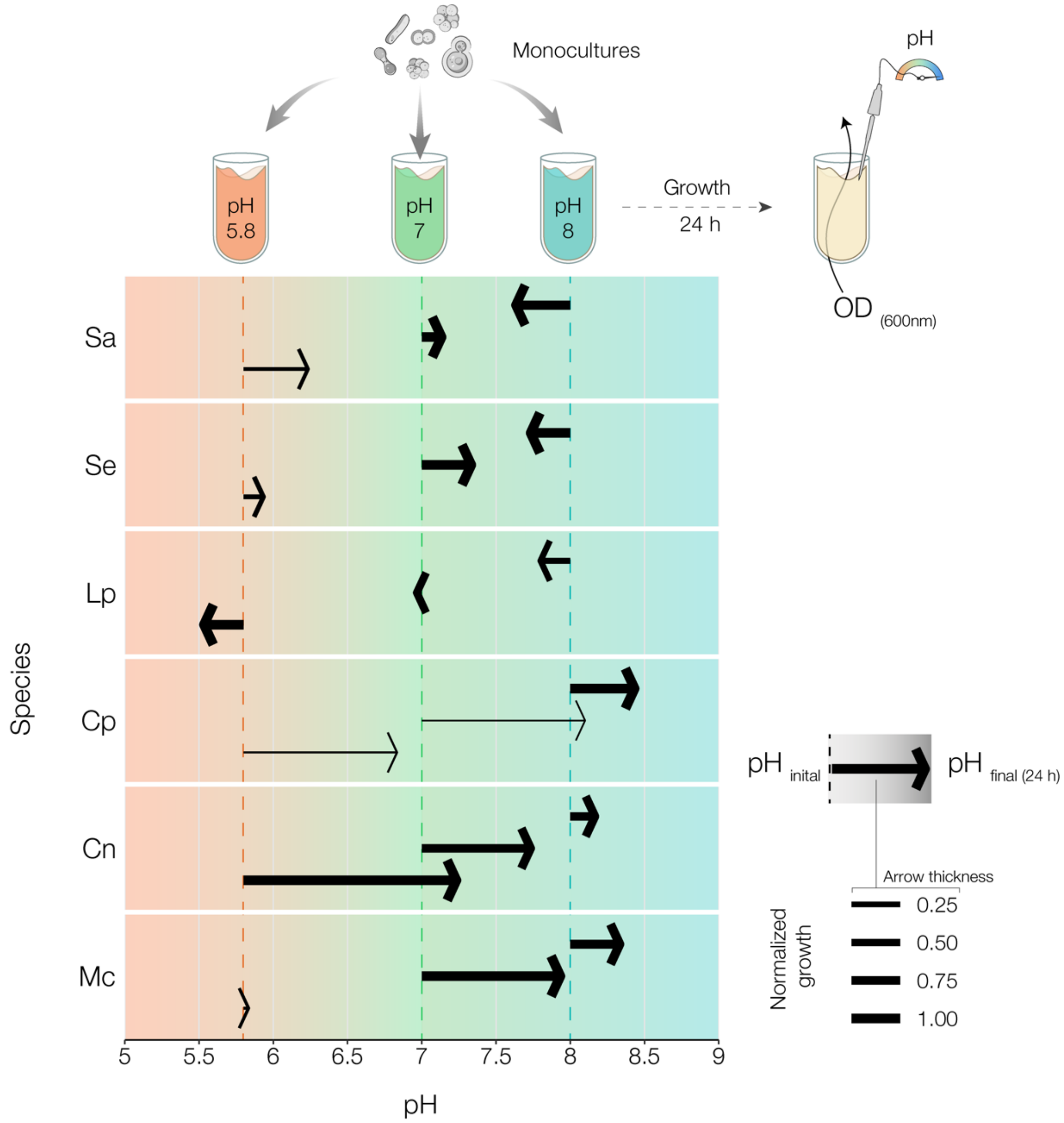

Supplementary Fig. 1| The majority of the six species increase the pH of culture media independently of the initial pH. Monocultures were grown in BHI at initial pHs: 5.8, 7 and 8. OD (600_{nm}) and pH were measured at the beginning of the experiment and after 24 h of growth. The length of arrows indicates pH changes induced by species at a given initial pH (average of four replicates). Arrow thickness is proportional to normalized growth and was calculated as the ratio between fold growth (OD_{24h}/ OD_{0h}) at a given initial pH and maximum species' fold growth across all initial pH conditions (average of four replicates). All focal species—Cn, Mc, Sa and Se—increase the pH of BHI media at initial pH 7, which was used in all the experiments described in the main text figures. Lp is the only species that lowers the pH in all conditions.

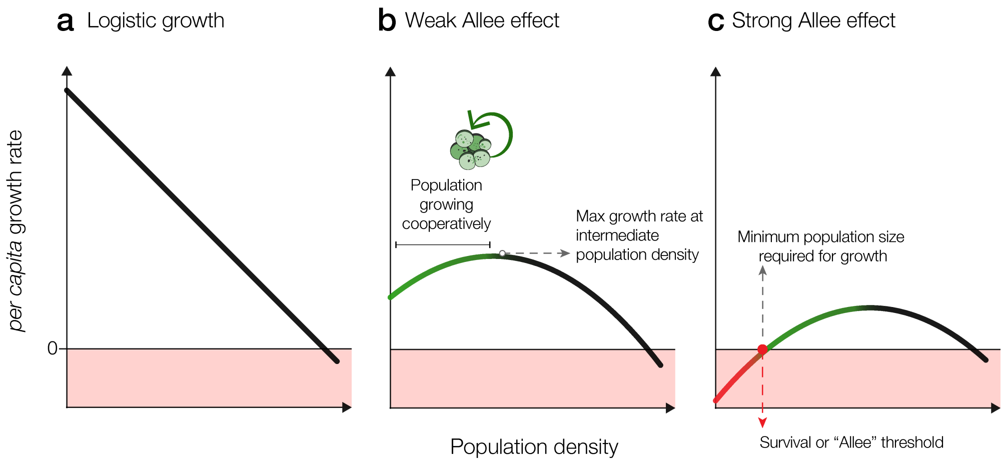

Supplementary Fig. 2| Cartoon illustrating different within-species population dynamics. a, Biological populations following logistic growth exhibit intraspecific competition, growing best at lower densities and experiencing a linearly decreasing *per capita* growth rate with population density. Conversely, some populations exhibit slower growth at low densities, growing best at intermediate densities due to intraspecific cooperation, known as the Allee effect. The Allee effect can vary in strength, being weak **(b)**, and therefore resulting in a reduced but positive growth rate at low density, or strong **(c)**, leading to a threshold abundance for survival below which the growth rate becomes negative, potentially leading the population to extinction.

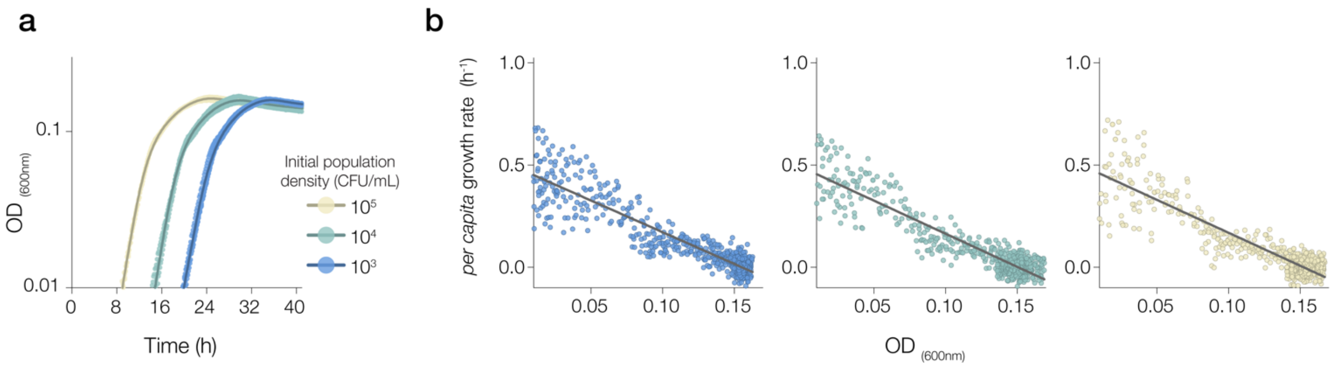

Supplementary Fig. 3| Cn exhibits logistic growth. a, To test whether the logistic growth model is a valid approximation for the growth of Cn, for which no Allee effect was experimentally observed (Figs. 2c and 4b), monocultures were grown at varying initial population densities over a period of 40 hours (OD_{600nm} measurements every ~10 min, minimum of three replicates for each condition). **b,** In the logistic growth model, the *per capita* growth rate decreases linearly with the population density. The data show the local *per capita* growth rate calculated as $ln\ OD_{t+\Delta t} - ln\ OD_t\ /\ \Delta t$**,** with the interval between measurements Δt=15min, as a function of the population density (OD_{600nm}). Lines show the results of a linear fit for populations initiated at 10^3 CFU/mL (slope = -3.105, R^2 = 0.8372), 10^4 CFU/mL (slope = -3.240, R^2 = 0.8452) and 10^5 CFU/mL (slope = -3.224, R^2 = 0.8059). For three different initial population densities, the data reveal an approximately linear decrease of the *per capita* growth rate with the population density, which confirms that Cn populations grow logistically.

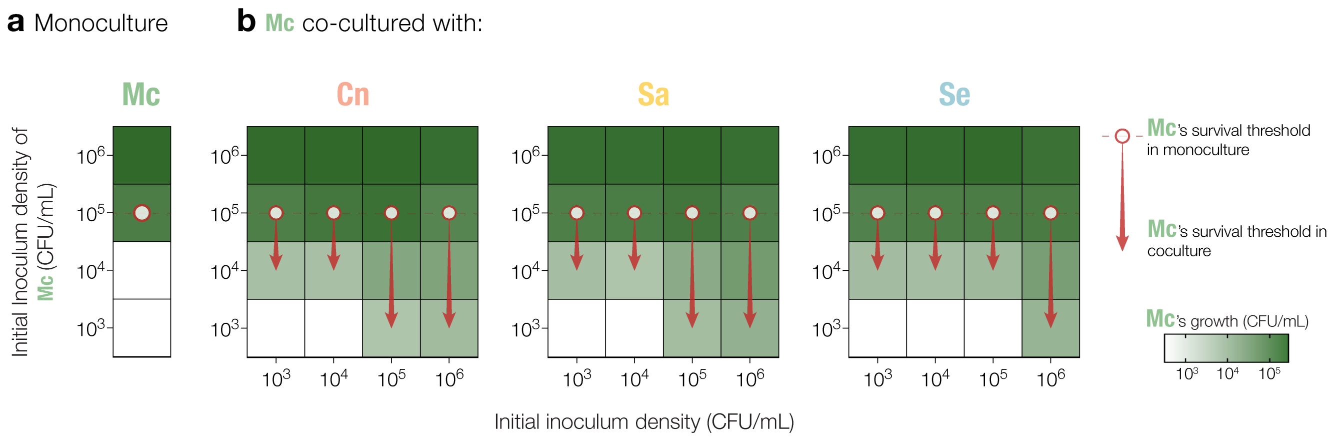

Supplementary Fig. 4| Mc's survival threshold is lower in coculture with all competitors. a, Mc monoculture inoculated in BHI at varying initial inoculum densities. CFU counting was performed after 6 h of incubation to determine population density (CFU/mL). No growth is observed below the survival threshold of Mc (~10^5 CFU/mL, white dot). **b,** Mc co-cultured with varying initial inoculum densities of Cn, Sa or Se. These three species facilitate the growth of Mc, lowering its survival threshold (arrow) in comparison to growth in monoculture. Data obtained from the average of 3 replicates for each condition.

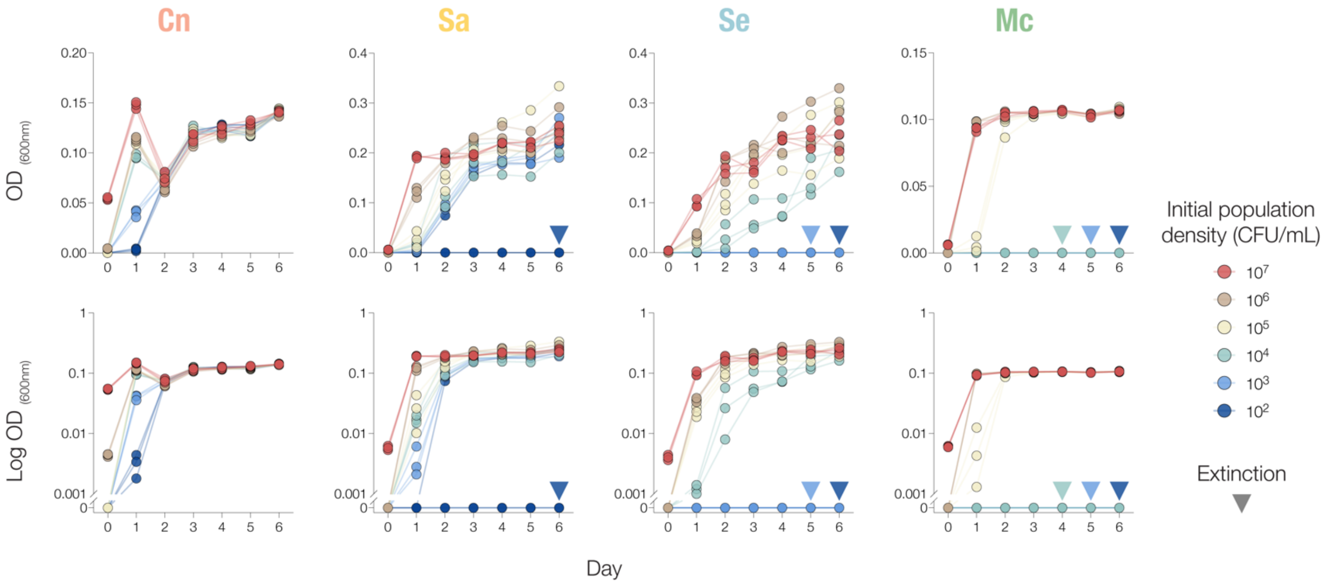

Supplementary Fig. 5| Population dynamics of species with the Allee effects exhibit survival thresholds under daily dilutions. We propagated monocultures at varying initial population densities over six days using a dilution factor of 100X. OD measurements are shown on linear (top) and log scales (bottom) for all focal species (n=3). Species subject to the Allee effect—Sa, Se and Mc—go extinct (inverted pyramid) when inoculated below critical (threshold) density. Replicate communities starting near the critical density may split, with some populations surviving and others going extinct.

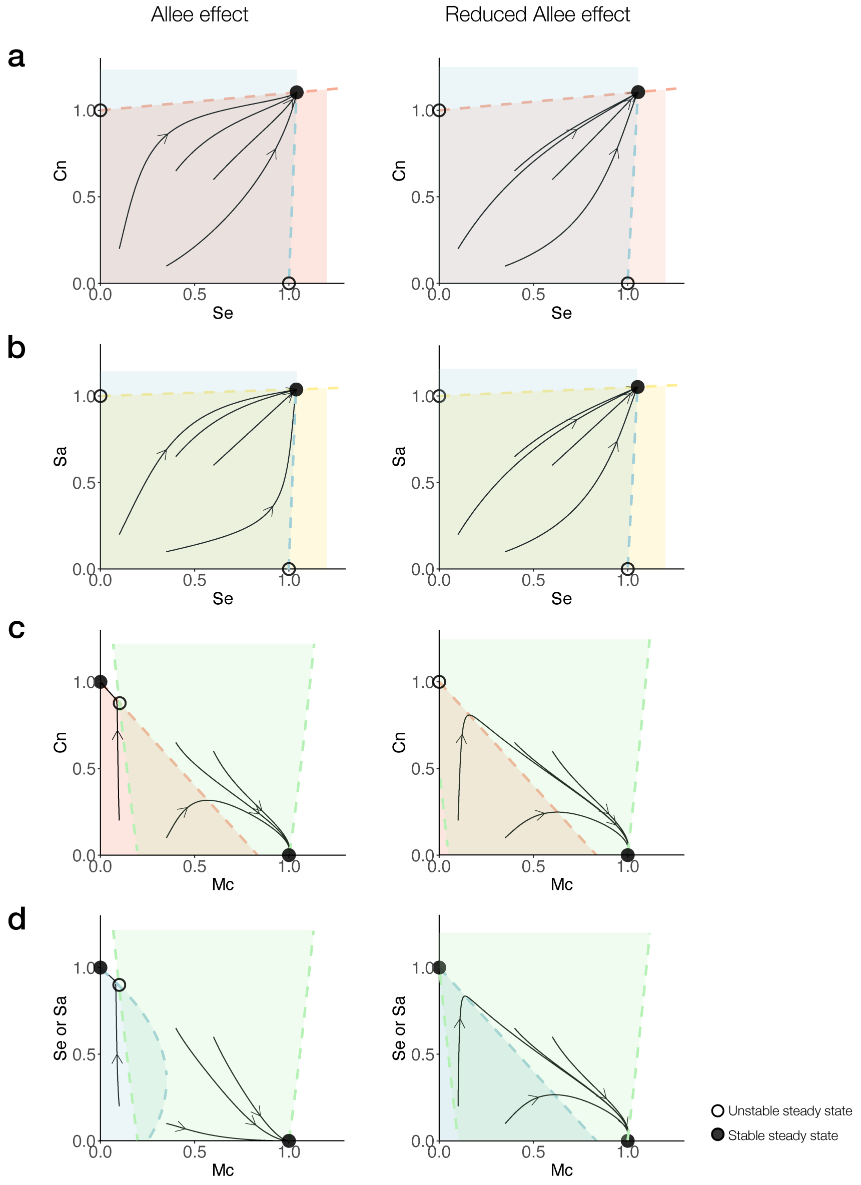

Supplementary Fig. 6| The LV model with cooperative interspecies interactions fails to capture the experimentally observed bistability driven by the weak Allee effect. Results from spent media experiments in Fig. 3b suggest that different pairs of species could exhibit facilitative interspecies interactions, which raises the question of how such facilitative interactions impact pairwise outcomes in the theoretical model. The panels show the nullclines, the stable (full dots) and unstable (empty dots) steady states, and representative trajectories (lines with arrows) in the phase space for different species pairs. The area under each nullcline, matched in color, indicates the region of positive *per capita* growth rate for the corresponding species. Here we chose the sign of interspecies interactions according to the outcomes of spent media experiments (Fig. 3b). For example, $a_{ij} < 0$ for i=Cn and j=Se, since we observed that the spent media of Se facilitates the growth of Cn. **a,** Phase space for Cn and Se, with Se exhibiting either a weak Allee effect (left) or logistic growth (right). The cooperative interspecies interactions play a stronger role in the outcome than the weak Allee effect, as evidenced by the relatively similar shape of the nullclines in the two panels. In the case of logistic growth, however, trajectories are

shorter and the species pair reaches the steady state faster. **b,** Phase space for Se and Se for both species under a weak Allee effect (left) or logistic growth (right). As in **a**, the system has a unique stable state in both cases—note that the interspecies cooperation enables the stable abundances to exceed the normalized carrying capacity. **c and d,** Under the strong Allee effect acting on Mc, the Allee threshold determines the competition outcome in addition to the interaction parameters. For a reduced Allee effect acting on Mc, Mc excludes Cn, while the system is bistable when Mc exhibits the strong Allee effect or competes against Se or Sa. Note that, due to the change in the interspecies interaction sign, the parabolic nullcline enables regions in which Mc can grow not matter how high the abundance of the other species is. Overall, the LV model does not predict bistability driven by a species' weak Allee effect if the interspecies interactions towards this species are cooperative. Our choice of using competitive interactions in the main text was based on: *i)* assessing interspecies interactions through experimental pairwise outcomes suggests that competition is present in co-cultures, ii) the LV model can lead to unrealistic, unbounded growth in the presence of cooperative interspecies interactions, and *iii)* spent media experiments are more suited to assessing intra-species interactions than inter-species interactions, since the spent media are generated by cells growing in the presence of the relevant interacting cell type (i.e. cells from the same species) in the case of intra-species interactions, but this is not the case in the case when inferring interspecies interactions—in the latter case, one monoculture produces the spent media that will be tested in a different species monoculture, potentially missing metabolites that are only produced in the presence of the other species[1].

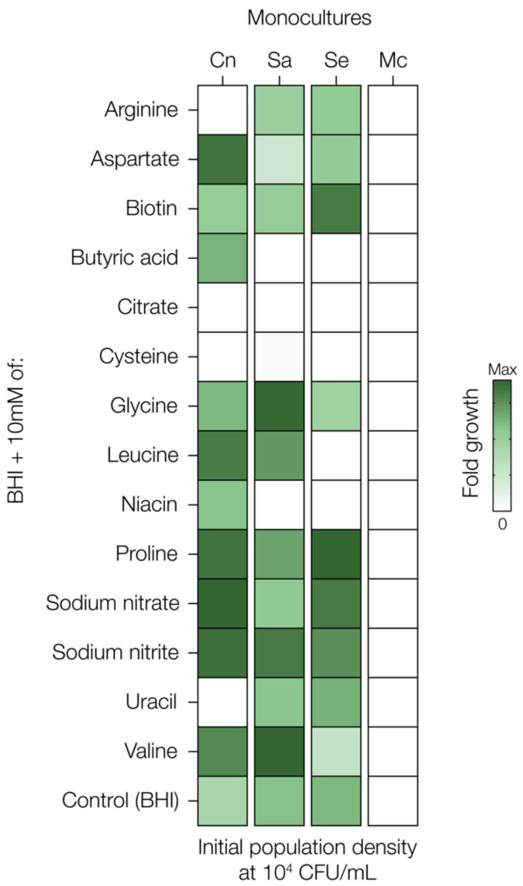

Supplementary Fig. 7| Biochemical screening reveals molecules capable of promoting species growth at initially low population density. We selected a set of molecules important to central metabolic pathways to supplement BHI (see Fig. 4a for additional molecules). Heatmap shows the monoculture fold growth (OD_{24h}/OD_{0h}, n=3) of focal species inoculated at low population density (10^4 CFU/mL) in supplemented BHI and control (BHI only, bottom). Maximum fold growth is scaled for each column relative to the maximum value observed for the corresponding species across conditions. Mc's growth below survival threshold (~10^5 CFU/mL) is only observed in glutamate supplementation (Fig. 4a).

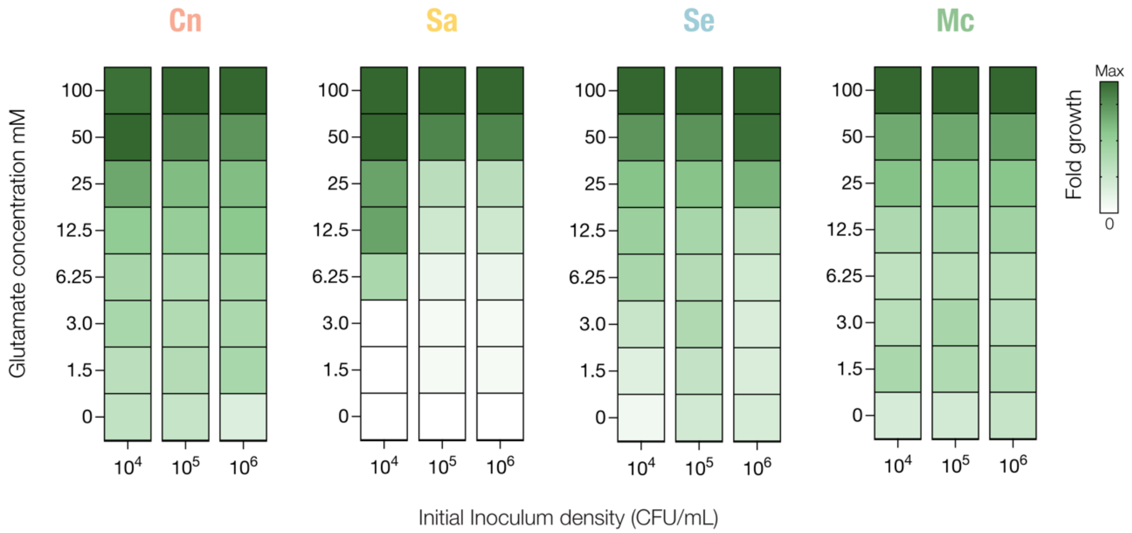

Supplementary Fig. 8| Dose response assay of increasing concentrations of glutamate shows maximum fold growth of species with the Allee effects at a concentration of 100mM. Heatmap shows the fold growth (OD_{24h}/OD_{0h}, n=3) of focal species in BHI supplemented with increasing concentrations of glutamate. Maximum fold growth occurred at a concentration of 100mM for Sa, Se and Mc. Maximum fold growth is scaled for each column according to initial inoculum density.

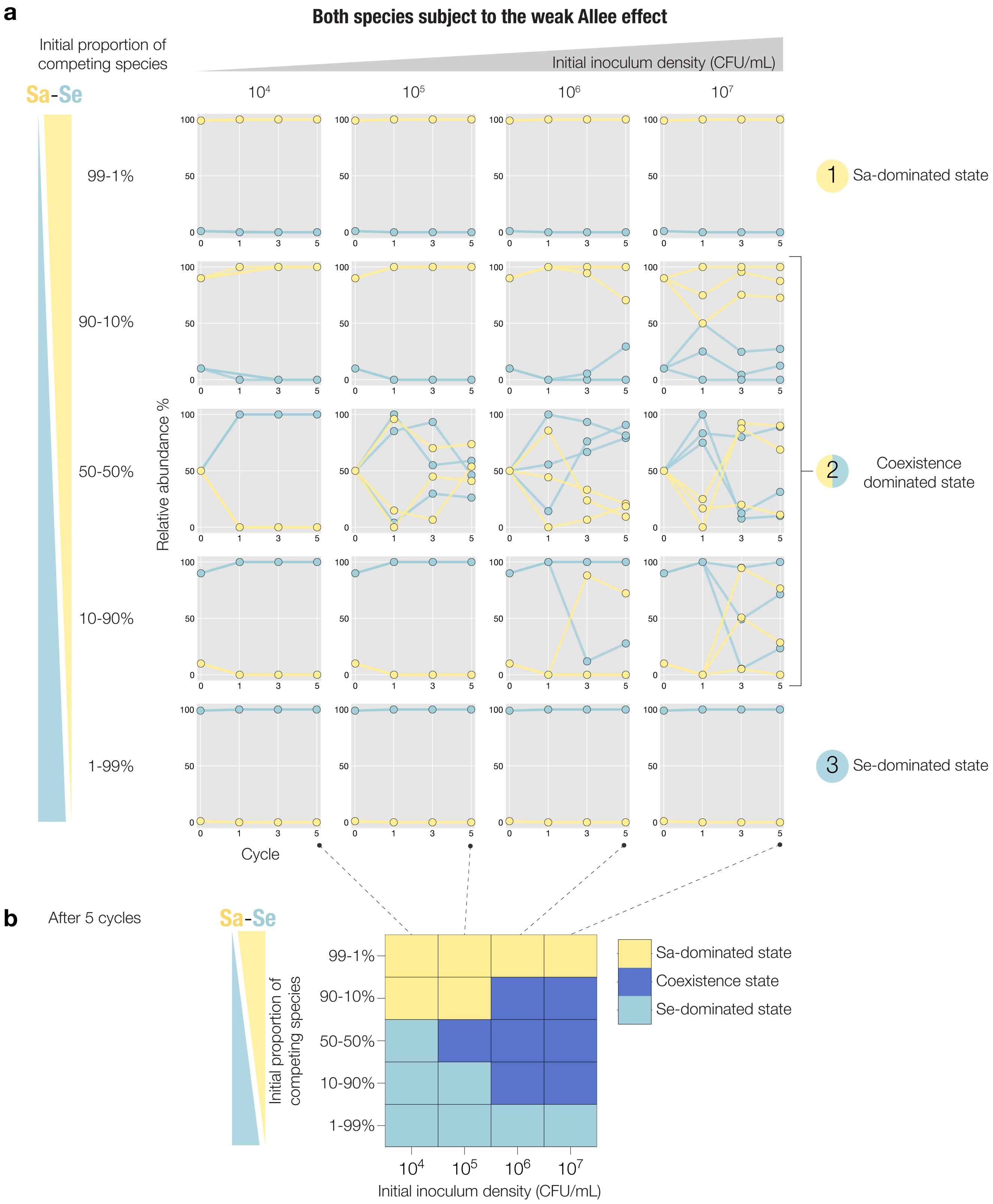

a
Both species subject to the weak Allee effect
Initial proportion of competing species
Sa-Se
Initial inoculum density (CFU/mL)
10^4
10^5
10^6
10^7
99-1%
90-10%
50-50%
10-90%
1-99%
Relative abundance %
Cycle
1 Sa-dominated state
2 Coexistence dominated state
3 Se-dominated state
b
After 5 cycles
Sa-Se
Initial proportion of competing species
Initial inoculum density (CFU/mL)
Sa-dominated state
Coexistence state
Se-dominated state

Supplementary Fig. 9| Tristability emerges in pairwise competition between Sa and Se, which exhibits both weak interspecies interactions and weak Allee effect. **a,** Results of additional experiments characterizing the competitions between Sa and Se mapped across various initial inoculum densities and different initial proportions of competing species. The non-canonical outcome of tristability is observed after 5 growth-dilution cycles, with initial inoculum densities ranging from 10^5 to 10^7 CFU/mL. The tristable outcome is composed of two states analogous to those of bistability (Sa-dominated state *1* and Se-dominated state *3*) and coexistence between species (state *2*). Time trajectories correspond to three replicates for each condition. Variability in replicates of the coexistence state indicates that tristability might be less stable. **b,** Phase diagram mapping the three stable states on the space of inoculum densities (average of three replicates shown in panel a).

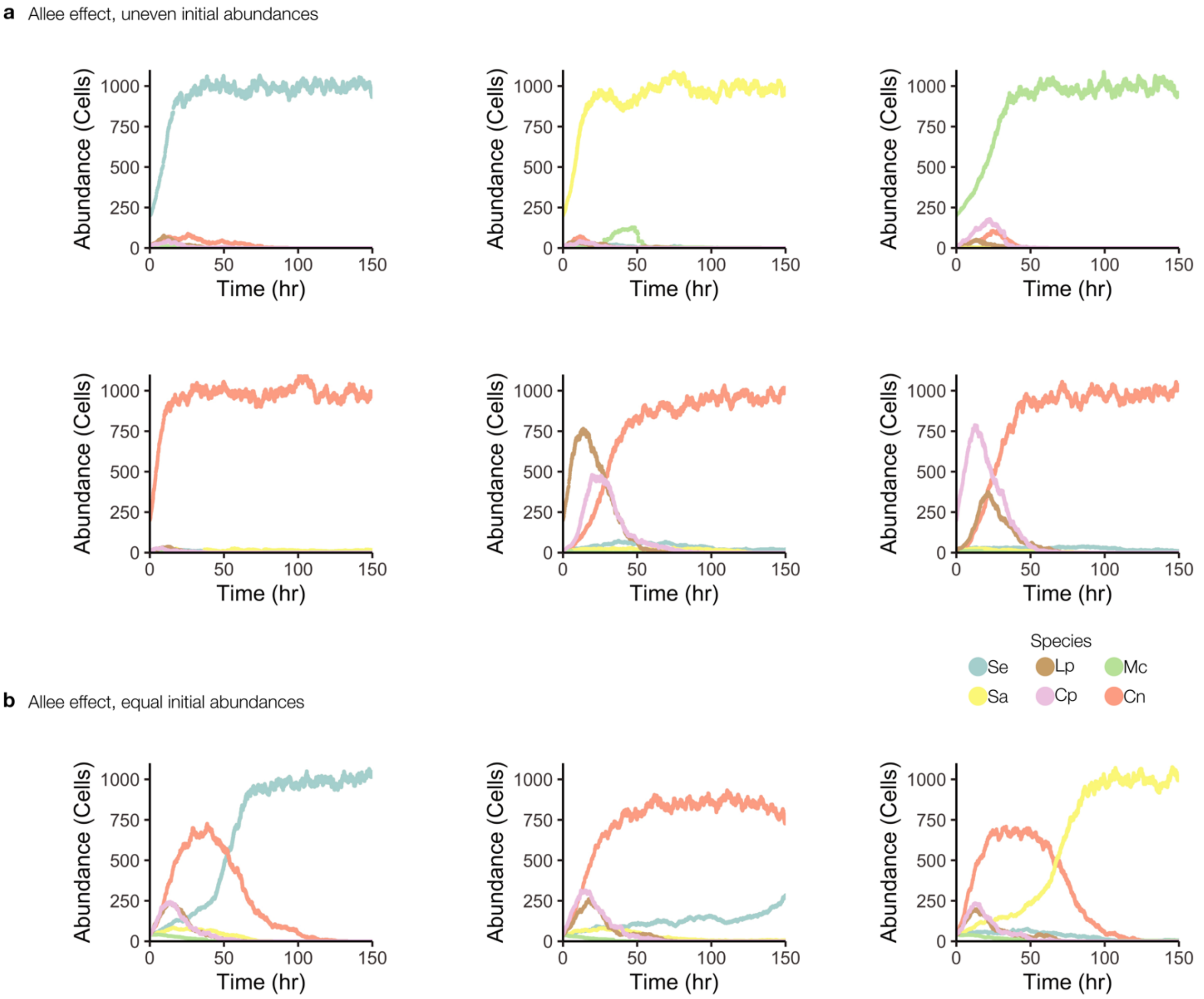

Supplementary Fig. 10| Sensitivity to stochastic fluctuations can lead to divergent community outcomes from the same initial conditions. The panels display representative results from simulations of the stochastic model considering competitive interspecies interactions, as well as a weak Allee effect acting on Sa and Se, and a strong Allee effect acting on Mc (Supplementary Text 1). **a,** Representative time series from stochastic simulations in which communities are initiated at uneven species abundances, where one species is initially dominant. For a fixed set of parameter values (Supplementary Text 1), the stochastic model recapitulates the four stable states observed experimentally. **b,** Starting from equal abundances for all six species, stochastic fluctuations can lead the community to reach alternative outcomes. The three panels show representative time series in which the community reaches the stable state dominated by Se (left), a case of slow dynamics in which Cn still coexists with Se (center) after 150 simulated hours (~6 days), and the reaching of the stable state dominated by Se (right). These three representative outcomes of stochastic simulations recapitulate the observed outcomes for communities inoculated at equal initial abundances (Fig. 1c), suggesting that sensitivity to stochastic fluctuations can lead the community to reach different outcomes from identical, or similar, initial conditions.

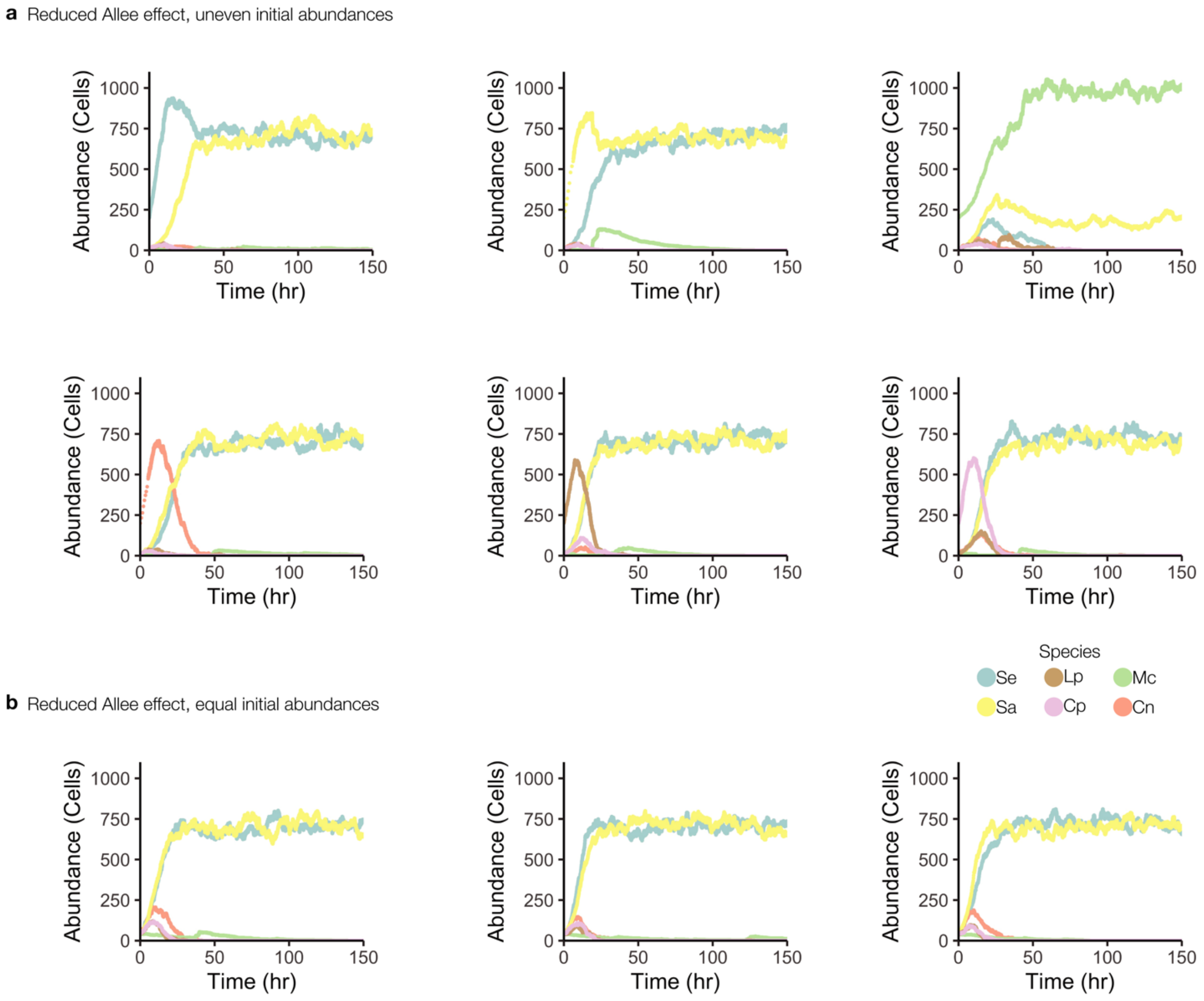

Supplementary Fig. 11| Reducing the Allee effect in stochastic populations recapitulates the observed changes in the number of alternative stable states. The panels display representative results from simulations of the stochastic model, which account for competitive interspecies interactions. This scenario considers only Mc subject to a strong Allee effect, while Sa and Se, as well as Cn, exhibit logistic growth (Supplementary Text 1). **a,** Representative time series from stochastic simulations in which communities are initiated at uneven species abundances for a reduced Allee effect scenario, which corresponds to the case of experimental cocultures under glutamate supplementation. Simulations recapitulate the reshaping of alternative stable states associated with the reduced Allee effect: the community reaches a stable state in which Se and Sa coexist under a wide range of different initial conditions. For the specific case in which Mc begins at relatively high density, the community reaches an alternative stable state in which Mc and Sa coexist. In these simulated scenarios, all the species grow logistically (Methods) except Mc, which is subject to the strong Allee effect (parameter value of the Allee threshold set to $c=0.02$). Parameter values are otherwise identical to those in Supplementary Fig. 10 (Supplementary Text 1). **b,** Starting from equal abundances for all six species, stochastic fluctuations

under the reduced Allee effect preferentially reach the stable state dominated by Se and Sa (this scenario is shown for direct comparison with Supplementary Fig. 10 although it was not experimentally tested).

Supplementary Text 1| Stochastic model and parameter values. To apply the optimized tau-leap method in the stochastic simulations (Supplementary Figs. 10 and 11), we used the equations of the theoretical model (Methods) in their non-normalized version. In the absence of an Allee effect, the dynamics of a given species *i* therefore reads:

$$\frac{dx_i}{dt} = r_i x_i \left(1 - \alpha_{ij} x_j / K_j\right) \qquad (4)$$

Where x_i stands for the absolute (non-normalized) abundance of species i, K_i for its carrying capacity, and the rest of the terms are analogous to those in the normalized version of the model (Methods).

For species that are subject to an Allee effect, the non-normalized version of the dynamics reads:

$$\frac{dx_i}{dt} = r_i x_i \left((x_i/K_i - a_i)(1 - x_i/K_i) - \alpha_{ij} x_j / K_j\right) \qquad (5)$$

Under the convention *i=1,2,3,4,5* and *6* corresponding to Se, Cn, Sa, Mc, Lp and Cp, respectively, we used the following parameter values:

$$r_i = 0.3\ hr^{-1}, \text{for } i = [1,6],$$

$$\boldsymbol{\alpha} = \begin{pmatrix} 1 & 0.4 & 0.4 & 1.2 & 0.1 & 0.1 \\ 1.05 & 1 & 1.2 & 1.6 & 0.3 & 0.3 \\ 0.4 & 0.4 & 1 & 0.8 & 0.1 & 0.1 \\ 0.1 & 0.1 & 0.1 & 1 & 0.1 & 0.1 \\ 1.3 & 1.3 & 1.3 & 1.5 & 1 & 0.1 \\ 1.3 & 1.3 & 1.3 & 1.5 & 0.1 & 1 \end{pmatrix}$$

In the simulations in Supplementary Fig. 10, we used the Allee effect strength values:

$$\boldsymbol{c} = (c_1, c_3, c_4) = (-0.3, -0.27, 0.05)$$

To model the reduction of the Allee effect under glutamate supplementation, in the simulations in Supplementary Fig. 11, only Mc (*i=4*) was subject to the Allee effect (with a reduced strength c_4=0.02).

It is worth noting that the parameter values used in the main text (Figs. 3d, 5a and 5b) were chosen in order to favor the visualization, in the corresponding phase planes, of the qualitative impact of the Allee effect on pairwise competitions outcomes. While the parameter values in the stochastic simulations (Supplementary Figs. 10 and 11) generate qualitatively the same pairwise outcomes presented in the main text, the set of parameter values used for the stochastic simulations enables a better recapitulation of the overall experimental outcomes, including the divergent outcomes for the 6-species community (Supplementary Fig. 10).

References:

1. Madsen, J. S., Sørensen, S. J. & Burmølle, M. Bacterial social interactions and the emergence of community-intrinsic properties. *Curr Opin Microbiol* **42**, 104–109 (2018).
