# Supplementary material for: Cooperative growth in microbial communities is a driver of multistability
Source: Nat Commun. 2024 Jun 3;15:4709. doi: 10.1038/s41467-024-48521-9 (PMC11148146; doi:10.1038/s41467-024-48521-9)
Supplement: Supplementary file 3 — Reporting Summary [file 41467_2024_48521_MOESM3_ESM.pdf]

nature portfolio

Corresponding author(s): William Lopes, Jeff Gore

Last updated by author(s): Apr 18, 2024

Reporting Summary

Nature Portfolio wishes to improve the reproducibility of the work that we publish. This form provides structure for consistency and transparency in reporting. For further information on Nature Portfolio policies, see our Editorial Policies and the Editorial Policy Checklist.

Statistics

For all statistical analyses, confirm that the following items are present in the figure legend, table legend, main text, or Methods section.

n/a	Confirmed	
☐	☒	The exact sample size (n) for each experimental group/condition, given as a discrete number and unit of measurement
☐	☒	A statement on whether measurements were taken from distinct samples or whether the same sample was measured repeatedly
☒	☐	The statistical test(s) used AND whether they are one- or two-sided<br>*Only common tests should be described solely by name; describe more complex techniques in the Methods section.*
☒	☐	A description of all covariates tested
☐	☒	A description of any assumptions or corrections, such as tests of normality and adjustment for multiple comparisons
☐	☒	A full description of the statistical parameters including central tendency (e.g. means) or other basic estimates (e.g. regression coefficient) AND variation (e.g. standard deviation) or associated estimates of uncertainty (e.g. confidence intervals)
☒	☐	For null hypothesis testing, the test statistic (e.g. F, t, r) with confidence intervals, effect sizes, degrees of freedom and P value noted<br>*Give P values as exact values whenever suitable.*
☒	☐	For Bayesian analysis, information on the choice of priors and Markov chain Monte Carlo settings
☒	☐	For hierarchical and complex designs, identification of the appropriate level for tests and full reporting of outcomes
☒	☐	Estimates of effect sizes (e.g. Cohen's d, Pearson's r), indicating how they were calculated

Our web collection on statistics for biologists contains articles on many of the points above.

Software and code

Policy information about availability of computer code

Data collection	The Tecan Infinite 200 Pro plate reader, along with proprietary software, was used to collect growth data, where OD 600 nm was measured. Colony-counting data was collected and stored using Microsoft Excel. The code to generate the phase diagrams and the stochastic simulations is available at https://doi.org/10.5281/zenodo.10975215.
Data analysis	Analyses were conducted in R, simulations were performed using the DeSolve package (version 1.40) and the GillespieSSa package (version 0.6.2). Analyses were also conducted using Prism (version 9+).

For manuscripts utilizing custom algorithms or software that are central to the research but not yet described in published literature, software must be made available to editors and reviewers. We strongly encourage code deposition in a community repository (e.g. GitHub). See the Nature Portfolio guidelines for submitting code & software for further information.

Data

Policy information about availability of data

All manuscripts must include a data availability statement. This statement should provide the following information, where applicable:

- Accession codes, unique identifiers, or web links for publicly available datasets
- A description of any restrictions on data availability
- For clinical datasets or third party data, please ensure that the statement adheres to our policy

Source data are provided with this paper

Research involving human participants, their data, or biological material

Policy information about studies with human participants or human data. See also policy information about sex, gender (identity/presentation), and sexual orientation and race, ethnicity and racism.

Reporting on sex and gender	n/a
Reporting on race, ethnicity, or other socially relevant groupings	n/a
Population characteristics	n/a
Recruitment	n/a
Ethics oversight	n/a

Note that full information on the approval of the study protocol must also be provided in the manuscript.

Field-specific reporting

Please select the one below that is the best fit for your research. If you are not sure, read the appropriate sections before making your selection.

[x] Life sciences [ ] Behavioural & social sciences [ ] Ecological, evolutionary & environmental sciences

For a reference copy of the document with all sections, see nature.com/documents/nr-reporting-summary-flat.pdf

Life sciences study design

All studies must disclose on these points even when the disclosure is negative.

Sample size	No calculation was used to determine the sample size. We selected six species that are particularly relevant to the respiratory tract due to their clinical and microbiological importance, including some recognized as "core members species". This selection approach aimed to capture a wide range of interactions among relevant species and enhance the ecological relevance and potential impact of our findings. The following species were used: Cryptococcus neoformans H99 (Cn, ATCC 208821), Corynebacterium pseudodiphtheriticum (Cp, ATCC 10700), Lactiplantibacillus plantarum (Lp, ATCC 8014), Moraxella catarrhalis (Mc, ATCC 25240), Staphylococcus aureus (Sa, ATCC 29213), and Staphylococcus epidermidis (Se, ATCC 14990).
Data exclusions	No data were excluded.
Replication	All experiments were performed at least three times under each experimental condition, and in each instance, the replications were successful.
Randomization	The experimental cocultures were inoculated with varying starting population densities and proportions of species.
Blinding	Blinding was not necessary for the experiments. The data were obtained by counting colonies with different morphologies, plated on selective/differential culture media when necessary.

Reporting for specific materials, systems and methods

We require information from authors about some types of materials, experimental systems and methods used in many studies. Here, indicate whether each material, system or method listed is relevant to your study. If you are not sure if a list item applies to your research, read the appropriate section before selecting a response.

Materials & experimental systems

n/a	Involved in the study	
[x]	[ ]	Antibodies
[x]	[ ]	Eukaryotic cell lines
[x]	[ ]	Palaeontology and archaeology
[x]	[ ]	Animals and other organisms
[x]	[ ]	Clinical data
[x]	[ ]	Dual use research of concern
[x]	[ ]	Plants

Methods

n/a	Involved in the study	
[x]	[ ]	ChIP-seq
[x]	[ ]	Flow cytometry
[x]	[ ]	MRI-based neuroimaging

Plants

Seed stocks	n/a
Novel plant genotypes	n/a
Authentication	n/a
